# Radon concentrations in kindergartens and schools in two cities: Kalisz and Ostrów Wielkopolski in Poland

**DOI:** 10.1007/s10967-012-2272-2

**Published:** 2012-10-04

**Authors:** Henryk Bem, Ewa Maria Bem, Joanna Krawczyk, Marcin Płotek, Sławomira Janiak, Daria Mazurek

**Affiliations:** Higher Vocational State School in Kalisz, ul. Nowy Świat 4, 62-800 Kalisz, Poland

**Keywords:** Indoor radon, Children exposure, Effective dose evaluation

## Abstract

Plastic PicoRad detectors with activated charcoal have been used for radon monitoring in local kindergartens and schools in two cities, Kalisz and Ostrów Wielkopolski, in the region of Greater Poland. Detectors were exposed for a standard time of 48 h during the autumn and winter of 2011 in 103 rooms (Kalisz) and 55 rooms (Ostrów Wlkp), respectively. The detectors were calibrated in the certified radon chamber of the Central Laboratory for Radiological Protection in Warsaw, Poland. The arithmetic and geometric means of indoor radon concentrations in the examined rooms were 46.0 and 30.3 Bq/m^3^ for Kalisz and 48.9 and 29.8 Bq/m^3^ for Ostrów Wlkp, respectively. The measured levels of the indoor radon concentrations were relatively low, since the main source of indoor radon for these low storey (max. three storeys) buildings is radon escaping from the underlying soil with a low ^226^Ra concentration (~15 Bq/m^3^). Therefore, the calculated annual effective doses from that source for the children in Kalisz and Ostrów Wlkp were also low 0.35 mSv.

## Introduction

Radon, in particular its longer living radionuclide ^222^Rn and its short lived daughters, is considered the second leading cause of lung cancers after tobacco [1]. Many worldwide epidemiological studies have been conducted in recent decades in order to confirm the association of the number of lung cancer cases with chronic exposure to indoor radon. As a result of these studies, summarized in the leading international organization elaborations, it is now one of the best documented associations [1–5]. Therefore, the International Committee for Radiological Protection (ICRP) recommendations emphasized the importance of controlling radon exposure in dwellings and work places arising from existing exposure situations [6].

Recent studies have also showed that children are more susceptible to radiation exposure than adults even for low doses obtained, for example, during CT examination [7] as well as those from slightly enhanced natural radiation [8]. However, until now there are no conclusive data on whether children are at greater risk than adults from radon. On the other hand, it is assumed that the lifetime attributable risks (LAR) for solid cancer incidence strongly depends on age of exposure, for example for children exposed to 0.1 Gy dose at age 10, the expected lung cancer incidence is twofold higher than that for people exposed to the same dose at age 30 [9]. Children also have longer latency periods for cancer developing as well as spending more time at home. For these reasons a special interest has been observed in indoor radon measurements in kindergartens and schools, and the majority of these results has been recently reviewed [10]. However, in Poland such measurements were carried out scarcely [11–14] and remarkably higher radon concentration exceeding 200 Bq/m^3^ have been observed in the Silesia region of Poland in areas affected by underground mining [13].

The aim of this study was to carry out a preliminary survey of radon levels in the kindergartens and schools in the two cities, Kalisz and Ostrów Wielkopolski, located in the southern part of the Greater Poland region, in the Fore-Sudeten monocline tectonic unit. Unfortunately, in the recently published data of the mean annual ^222^Rn concentration in homes for the whole of Poland, this region had not been taken into consideration [15].

We have previously proved that the PicoRad detector method using plastic scintillation vials with charcoal for Rn adsorption and liquid scintillation finishing is very convenient for large scale surveillance of Rn in dwellings [14, 16–19]. The temperature fluctuations during a standard 48 h detector exposure time can be easily corrected (if necessary) by a proposed computational program [18] and that exposure time is sufficient to average the diurnal variations in radon concentrations in the examined rooms [19].

## Method of measurements

Commercially available plastic liquid scintillation vials with charcoal PicoRad were purchased from AccuStar, a Spruce Company (USA). The vials were exposed for 48 h in the chosen rooms of all the kindergartens, play-schools and schools in Kalisz (103 rooms) and Ostrów Wielkopolski (55 rooms) during the period from 15th of October to 15th of December 2011. As the vast majority of these building were equipped with central heating systems, the room temperatures during the time of the exposition were relatively constant: 22 ± 2 °C. The radon adsorbed in charcoal of the returned vials after exposure was eluted with 10 ml of liquid scintillation cocktail containing: 8 g/dm^3^ butyl-PBD and 0.3 g/dm^3^ dimethyl POPOP in the mixture: 90 % toluene plus 10 % (v/v) methanol. The activities of the eluted ^22^Rn and its four short-living daughters were measured (at least 8 h from the beginning of elution) in the fixed channel of the liquid scintillation counter LKB Rack beta 1219SM for 1 h for each sample. The details of the measuring conditions as well as the quality assurance and the accuracy of the method were described elsewhere [17]. For these sets of experiments the detectors were calibrated in the certified radon chamber of the Central Laboratory for Radiological Protection in Warsaw, Poland. The five calibration vials were exposed for 48 h in that chamber at constant ^222^Rn concentration equal to 297 Bq/m^3^ at temperature 22 ^°^C. The average so-called calibration coefficient *K* of the method was calculated from the formula:

1 $$ K = \sum I_{{i}} / 5\cdot C_{\text{Rn}} $$

where Σ *I*_*i*_ is the sum of the measured net activities of five calibration vials (cpm), *C*_Rn_ is the ^222^Rn activity in the calibration chamber (Bq/m^3^).

In these experiments the average value of calibration coefficient *K* = 0.557 ± 0.031 was used. Indoor radon concentrations were calculated from the formula:

2 $$ C_{\text{Rn}} = \, K^{ - 1} \cdot I \cdot { \exp }\left( {\lambda \cdot t_{\text{d}} } \right) \, \left( {{\text{Bq}}/{\text{m}}^{3} } \right) $$

where *I*, activity of the ^222^Rn and its decay products in cpm; λ = 7.55 × 10^−3^ h^−1^, decay constant for ^222^Rn, *t*_d_, delay time in h, from the end of the vial exposition to an activity measurement.

## Results and discussion

**Fig. 1:**
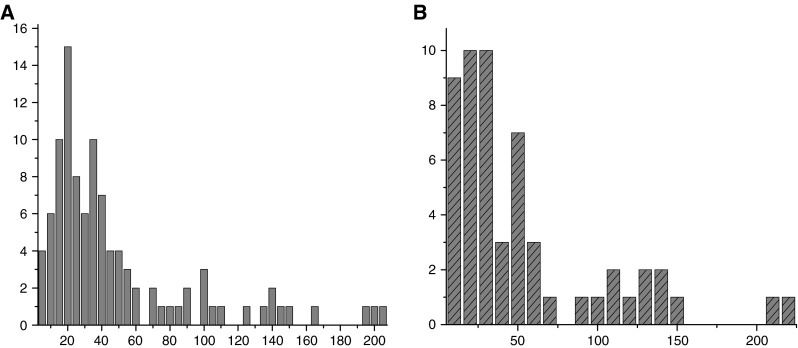
Distribution of radon concentrations in Kalisz (**A**) and Ostrów Wielkopolski (**B**) area

The distributions of radon concentrations for all measured rooms in Kalisz and Ostrów Wielkopolski kindergartens and schools are shown in Fig. 1a, b, respectively. Since the number of examined rooms in Kalisz (*N*_K_ = 103) was almost twofold higher than those in Ostrów Wlkp (*N*_O_ = 55), the Rn concentrations intervals were chosen as equal to 5 Bq/m^3^ for Kalisz and 10 Bq/m^3^ for Ostrów Wlkp. In both towns the observed radon levels are relatively low: from 5 Bq/m^3^ (lower limit of detection) up to 194.4 and 216.8 Bq/m^3^ in Kalisz and Ostrów Wlkp, respectively.

The remaining radon concentration distribution parameters for these distributions are shown in Table 1.

**Table 1:** Parameters of ^222^Rn concentration distributions in Kalisz and Ostrów Wielkopolski kindergartens and schools

Parameter	Kalisz	Ostrów Wielkopolski
Number of flats	103	55
Arithmetic mean (Bq/m^3^)	46.0	48.9
Arithmetic mean standard deviation (Bq/m^3^)	45.0	49.0
Geometric mean (Bq/m^3^)	30.3	29.8
Geometric standard deviation	2.48	2.86
Median (Bq/m^3^)	31.5	26.9
Minimum concentration (Bq/m^3^)	5^a^	5^a^
Maximum concentration (Bq/m^3^)	194.4	216.8

^a^Corresponds to the lower limit of detection for used method

The calculated arithmetic and geometric means of radon concentrations in these cities are comparable: 46.0 and 30.3 Bq/m^3^ for Kalisz and 48.9 and 29.8 Bq/m^3^ for Ostrów Wlkp. However, as is evident from these figures the results do not well fit to typical log-normal distributions, and corresponding values of geometric mean standard deviations are relatively high.

The observed average arithmetic and geometric values for radon concentrations are higher than those observed for the city of Lodz in central Poland: 17.9 and 13 Bq/m^3^ [13], respectively. There are no experimental data on the ^226^Ra concentrations in surface soil in these two cities. However, according to the Radiological Atlas of Poland [20] ^226^Ra concentrations in this region are only slightly higher than those for the Lodz area (average ~15 Bq/kg). Therefore, these differences can be explained by the fact that the overwhelming majority (80 %) of the kindergartens, play schools and schools in these two cities are two storey buildings and the major contribution to the total indoor radon activity comes from radon escaping the underlying soil. It is worth noticing that the values of the geometric mean concentrations and medians are very close to each other in both cities and therefore, the geometric mean values should be taken for the effective dose calculations.

## Effective dose calculation for children

The total annual effective dose *E*_Rn_ caused by inhalation of the radon and its decay product can be calculated from the following formula;

3 $$ E_{\text{Rn}} = {\text{ DCF }}\left( {C_{\text{in}} \cdot t_{\text{in}} \cdot F_{\text{in}} + \, C_{\text{ot}} \cdot t_{\text{ot}} \cdot F_{\text{ot}} } \right) $$

where DCF is a radon dose conversion factor [mSv/(Bq h m^−3^)], *C*_in_ and *C*_ot_—are annual average indoor and outdoor radon concentrations, respectively (Bq/m^3^), *t*_in_ and *t*_ot_ are average annual indoor and outdoor exposures, respectively (h), *F*_in_ and *F*_ot_ are indoor and outdoor radon-daughters equilibrium factors, respectively.

The dose conversion factor values for radon inhalation has been recommended by different organizations on the base of epidemiological studies concerning the risks the lung cancer from residential and occupational exposure to radon, as well as upon the dosimetric models using the ICRP Human Respiratory Tract Model (HRTM). The dosimetric models assume that nearly the entire lung dose arises from the inhalation of the radon progenies deposited in the respiratory airways of the lung. As a result of these two different ways of dose evaluation, one could observe some discrepancy in the recommended DCF values [21]. However, the ICRP on the basis of the recent epidemiological data revised the lung cancer risk estimates caused by Rn inhalation to the higher value of 8 × 10^−10^ Bq h m^−3^, and consequently to the higher value of DCF = 10 nSv/Bq h m^−3^ [2]. This value is very close to the 9 nSv/B Bq h m^−3^ recommended by UNSCEAR [5].

The annual average indoor radon concentration *C*_in_ in examined rooms can be calculated by multiplying the mean concentration determined by us in the October–December period by the appropriate seasonal correction factor resulting from seasonal fluctuation of indoor radon in dwellings for that region of Poland [22]. The average value of this correction factor for buildings with basements in this area was 0.96.

As previously determined for buildings in Central Poland, the average equilibrium factor for radon and its daughters *F*_in_ = 0.6 [19].

The annual effective dose for children during their stay in the kindergartens and preschools in these two cities can be calculated assuming their 2,000 h annual indoor stay in these buildings. Therefore, introducing the above described values, one can obtain:

$$ E = 10 \times 30  \times 0.96  \times 0.6 \times 2,000 = 3.5 \times 10^{5}\,\text{nSv} = 0.35\,\text{mSv} $$

Such calculated average annual effective dose for children from radon and its daughter’s inhalation in kindergartens and preschools can be compared to the annual average effective dose from all natural sources of radiation, which in Poland is equal to 2.43 mSv [20]. However, one should take into account fact that it is only part of their total inhalation doses, not including indoor exposure in their houses and outdoor exposure to radon and its daughters according to Eq. (3). Moreover, for the highest observed indoor radon concentrations ~200 Bq/m^3^, the corresponding annual dose during the children’s stay in these rooms will exceed 2.3 mSv. Taking into account the at least twofold higher risk of radiation to children than for adults mentioned above, such exposure needs a proper action to mitigate the Rn concentrations in these buildings. Although the ICRP recently revised the upper value of the so called reference level for radon gas in dwellings from 600 to 300 Bq/m^−3^ [2], there is still a lack of any international recommendation for permissible indoor radon concentrations in buildings used by children.
